# Critical illness following kidney transplant: a case of xanthogranulomatous cholecystitis in a patient with multiple comorbidities

**DOI:** 10.1093/jscr/rjaf814

**Published:** 2025-10-14

**Authors:** Peter C Lampman, Kristin E Trone, Lindsey Loss, Reynold Henry

**Affiliations:** Department of Surgery, Oregon Health and Sciences University, 3181 SW Sam Jackson Park Rd., Portland, OR 97239, United States; Department of Surgery, Oregon Health and Sciences University, 3181 SW Sam Jackson Park Rd., Portland, OR 97239, United States; Department of Surgery, Oregon Health and Sciences University, 3181 SW Sam Jackson Park Rd., Portland, OR 97239, United States; Department of Surgery, Oregon Health and Sciences University, 3181 SW Sam Jackson Park Rd., Portland, OR 97239, United States

**Keywords:** kidney transplant, subcapsular hematoma, xanthogranulomatous cholecystitis, end-stage renal disease, critical care

## Abstract

We report the case of a 63-year-old female with significant comorbidities, including end-stage renal disease secondary to type 2 diabetes mellitus, who underwent a left kidney transplant. Post-operatively, she developed complications, including subcapsular hematoma, acute hypoxic respiratory distress, and bacteremia complicated by xanthogranulomatous cholecystitis. Despite aggressive medical interventions, her condition deteriorated, and comfort care measures were initiated. This case underscores the complexities and challenges in managing critically ill post-transplant patients and highlights the importance of a high index of suspicion of less common infections in this patient population.

## Introduction

Kidney transplantation offers a life-saving treatment for patients with end-stage renal disease (ESRD). However, it carries risks of perioperative complications. This report describes a challenging case involving multiple post-operative complications, underscoring the importance of comprehensive and collaborative care in managing complex patients.

## Case presentation

**Figure 1:**
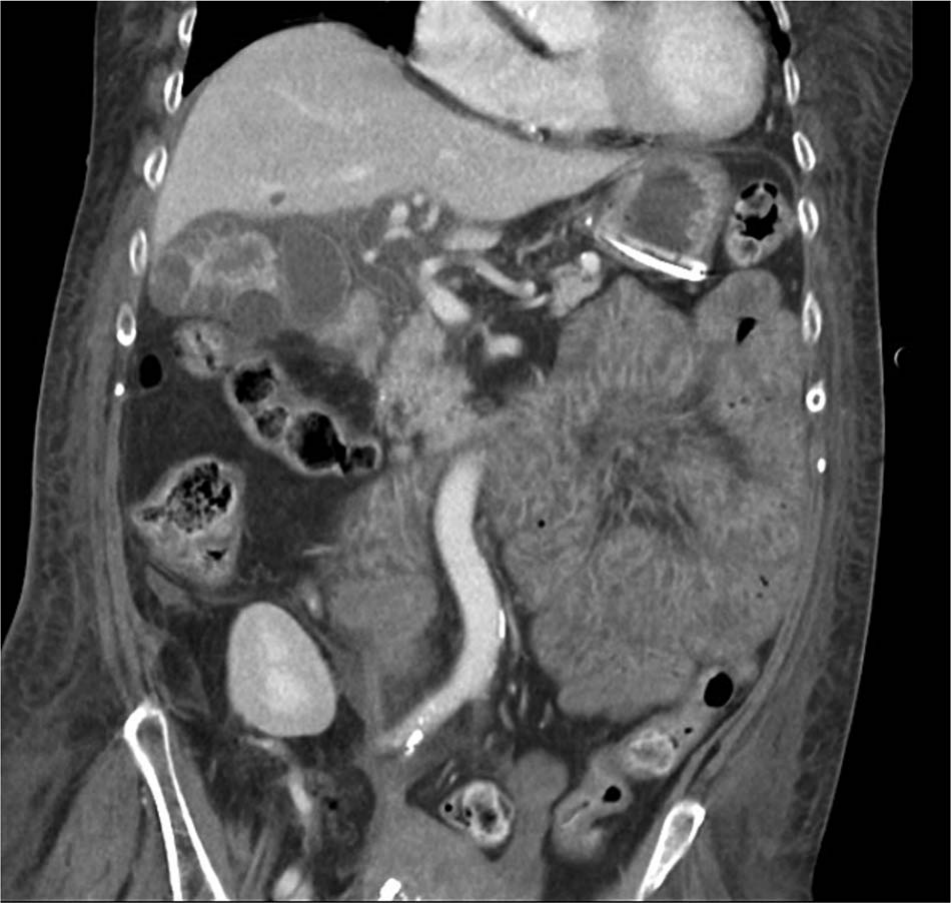
CT chest abdomen pelvis with IV contrast coronal view. The common bile duct is visible and without dilation, note multiple intramural hyperattenuating nodules and gallbladder wall hyperenhancement. This image also shows diffusely edematous jejunum indicative of systemic hypoperfusion.

**Figure 2:**
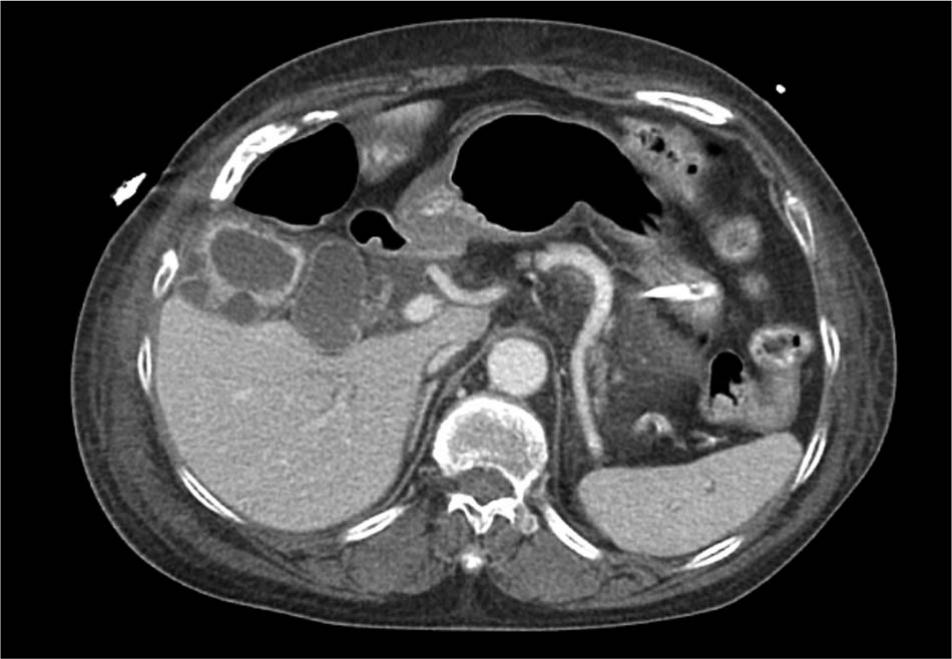
CT chest abdomen pelvis with IV contrast axial view. Gallbladder wall hyperenhancement and multiple intramural hyperattenuating nodes are again apparent in axial imaging.

A 63-year-old female with a history of developmental delay, hypertension, hyperlipidemia, paroxysmal atrial fibrillation, hyperparathyroidism status post-parathyroidectomy, and ESRD on intermittent hemodialysis who presented for a scheduled left kidney transplant. Her post-operative course was complicated by subcapsular hematoma resulting in significant blood loss and requiring resuscitation in the trauma-surgical intensive care unit (TSICU); acute hypoxic respiratory distress on post-operative day (POD) 2, necessitating reintubation and escalating vasopressor support; and bacteremia and xanthogranulomatous cholecystitis identified on POD 3, confirmed by imaging (Figs 1 and 2) and blood cultures positive for *Klebsiella pneumoniae* and *Enterobacter cloacae* with Cefotaxime-Munich (CTX-M) resistance.

Due to her hemodynamic instability interventional radiology was consulted to place a percutaneous cholecystostomy at bedside, which was successful. Despite these measures, her condition continued to decline with refractory hypotension and hypoxemia. The family elected for comfort care, and the patient passed away on POD 4.

## Discussion

This case highlights the interplay of an extremely rare infection in a high-risk transplant patient.

Xanthogranulomatous cholecystitis (XGC) is a rare and severe inflammatory condition of the gallbladder, characterized by the presence of xanthogranulomatous inflammation and destruction of gallbladder tissue. The exact etiology of XGC is not fully understood, but it is often associated with chronic infection, obstruction, or stones within the biliary tract, leading to recurrent inflammation and tissue destruction. Its presentation can mimic malignancy both clinically and radiologically, making timely diagnosis challenging [1].

In XGC, prolonged inflammation results in fibrosis and lipid-laden macrophages infiltrating the gallbladder wall, which can extend into adjacent structures [2]. This process can contribute to complications such as perforation, abscess formation, or fistula development. In the case described, the patient’s XGC was compounded by severe systemic infection and bacteremia, further complicating her critical illness. The bacteremia was caused by multidrug-resistant pathogens, including *K. pneumoniae* and *E. cloacae*, underscoring the vulnerability of immunocompromised transplant patients to atypical and aggressive infections.

XGC is typically diagnosed through imaging and histopathological examination. In this case, computed tomography (CT) revealed findings consistent with XGC, including gallbladder thickening and inflammatory changes [3]. However, definitive diagnosis was confirmed by blood cultures and the clinical course. The overlapping features with cholecystitis and biliary tract malignancies pose diagnostic challenges, particularly in acutely ill patients. In transplant recipients, where symptoms may be atypical and infection progresses rapidly, clinical suspicion and early intervention are paramount.

Management of XGC in critically ill patients, such as this case, is complex and requires a multidisciplinary approach. Treatment typically includes antibiotics tailored to culture results, with escalation to meropenem in response to CTX-M resistance. Surgical or interventional radiology approaches are also critical, with percutaneous cholecystostomy serving as a temporizing measure in this unstable patient. Despite these interventions, the patient’s hemodynamic instability and refractory respiratory failure reflected the severity of systemic involvement.

Early recognition of XGC in transplant recipients presenting with sepsis and gallbladder pathology is essential. Aggressive source control through minimally invasive interventions can be life-saving in critically ill patients. The prevalence of resistance genes like CTX-M underscores the need for rapid diagnostic techniques and targeted antimicrobial therapy. The immunocompromised state of transplant recipients necessitates heightened vigilance for atypical infections and inflammatory conditions.

The case emphasizes the importance of multidisciplinary collaboration involving transplant surgery, critical care, infectious disease, and interventional radiology teams. While XGC remains a rare diagnosis, its potential to cause severe systemic complications in vulnerable populations like transplant recipients highlights the need for a high index of suspicion and proactive management strategies. Further research into the unique pathophysiological mechanisms of XGC in immunocompromised patients could improve diagnostic accuracy and outcomes.

## Conclusion

This case illustrates the challenges of managing complex transplant recipients and emphasizes the need for early recognition of complications, aggressive resuscitative measures, and empathetic communication with patients’ families.
